# Bismuth as a Dressing for Wounds

**Published:** 1886-08-20

**Authors:** 


**Bismuth as a Dressing for Wounds.**—M. Gosselin and M. Herat (Progr. Med.) have been studying the mode of action of subnitrate of bismuth upon wounds. It is known that this drug has the effect of diminishing the oozing of blood after an operation. Although, of itself, it has no coagulating power, it acquires such a property by virtue of the disengagement of nitric acid upon the mouths of the capillaries. Moreover, it has an astringent action, due at the same time to the nascent nitric acid, and to the oxide of bismuth, a germicidal effect, and a special sedative operation. The subnitrate should be preferred, as the other salts have not, of course, the coagulating and constrictive power that is due to the acid. It may be used either in powder or by irrigation in the proportion of 1 to 50.—*New England Med. Monthly.*

THE address in medicine before the British Medical Association, which was to have been delivered by the late Dr. Austin Flint, will be given by another American, Dr. John S. Billings, of the Uuited States Army.—*Physician and Surgeon.*

